# Systematic screening of infection prevention policies for equity impacts

**DOI:** 10.1017/ash.2025.16

**Published:** 2025-03-14

**Authors:** Caitlin L. McGrath, Yasaman Fatemi, Thérèse Mirisola, Tanya Ferreira, Adrienne D’Alo, Victoria J.L. Konold, Alicia Tieder, Ashley Stratton, Matthew P. Kronman, Danielle M. Zerr

**Affiliations:** 1 Department of Pediatrics, Division of Pediatric Infectious Diseases, University of Washington School of Medicine, Seattle, WA, USA; 2 Seattle Children’s Research Institute, Seattle, WA, USA; 3 Seattle Children’s Hospital, Seattle, WA, USA; 4 Seattle Children’s Hospital Center for Quality and Patient Safety, Seattle, WA, USA; 5 Seattle Children’s Center for Diversity and Health Equity, Seattle, WA, USA

## Abstract

We reviewed infection prevention policies using an adapted Equity Impact Assessment tool. Thirty-one percent of policies had substantial potential to impact marginalized groups and create or sustain inequities, and most lacked existing equity considerations. Systematic policy review for equity implications can result in actions to improve care and quality.

## Introduction

The impact of racism, bias, and other structural factors resulting in inequitable outcomes throughout medical care is well-documented.^1^ Outcomes that reflect hospital quality and safety are no exception, with inequitable outcomes noted for various healthcare-associated infections.^2,3^

Infection prevention (IP) programs play integral roles in ensuring patient safety and quality care, and policies used within healthcare organizations are influenced and utilized by IP programs to standardize and guide practice. However, some policies may inadvertently uphold institutional racism and discrimination.^4^ Our institution utilizes an Equity Impact Assessment (EIA) tool during new policy creation or periodic existing policy updates or reviews to identify, reduce, eliminate, and prevent inequities in care.^5^ EIAs, and related tools including health equity impact assessments and racial equity impact assessments, are used in various settings such as public health, public policy, and education.^6–8^ We aimed to review our existing IP policies using an equity screening tool to determine both the frequency with which IP policies had the potential for substantial equity impacts and to prioritize our own future work in updating our policies to address those potential impacts.

## Methods

Our freestanding children’s hospital serves as a regional pediatric healthcare anchor institution and referral center. The IP program at our hospital is multidisciplinary and includes infection preventionists as well as pediatric infectious diseases physician medical directors. Our hospital implemented the institutional EIA tool^5^ in 2021 and began requiring its use for policy creation or updates of organizational policies in 2022. This has been facilitated by our Center for Diversity and Health Equity consultants, who partner with teams throughout the organization to improve equitable care.

Given that a) existing organizational IP policies would be updated over the course of several years and not receive a review using the institutional EIA tool until that time, and b) the IP program also uses internal guidelines that would not undergo a review using the institutional EIA tool, we created an “IP Equity Screen” (Table 1). The IP Equity Screen was adapted from the institutional EIA tool^5^ via an iterative process with equity and quality improvement content experts (AS, TK). The goal was to highlight key questions to use when considering policy topics, subjects, and impacts and to provide an initial method of screening IP policies to determine where to prioritize and measure equity work within our program.

**Table 1.** Equity screening tool for infection prevention policies (infection prevention equity screen)

Screening Question	Example
1. Are there **known inequities and/or disparities** related to the subject of the guideline, from institutional data or literature search?	Known history of disparities in which populations are most affected by multi-drug resistant organisms (MDROs).
2. Are any **historically disenfranchized or underrepresented groups** likely to be most affected by and concerned with this guideline? a. Are members from the affected groups meaningfully engaged in guideline development or revision?	Known impacts of language of care and access to interpreter services on family engagement around discussions of TB isolation.
3. Could this guideline result in potential **adverse impacts or could unintended consequences** result from this guideline? a. What are they? How will they be monitored for? How might we prevent this?	Visitor restrictions such as seasonal restriction of siblings designed to protect patients from communicable diseases may have unintended negative consequences that should be considered, such as family impacts when alternative childcare is not available.

We reviewed current institution-wide policy documents (n = 119) related to or managed by Infection Prevention at our hospital using the IP Equity Screen. Each policy topic and text were assessed for its potential to create or sustain inequities for patients, families, or staff. Initial policy review was independently performed by two physician IP medical directors (CM, YF) after training on the tool with a health equity content expert; disagreements were resolved by consensus. Policies were considered to screen in when the answer to one of more questions of the IP Equity Screen (Table 1) was “yes.” The questions were designed to help our team consider or identify known inequities, potential inequitable impacts on systemically underserved groups, or potential unintended consequences of policies.

Policies determined to have potential inequities were examined for any language to suggest that equity considerations had been incorporated into the existing policy. The presence of equity considerations was defined as any explicit mention of disparate impact of the policy on underrepresented groups or mitigation of such effect.

## Results

Of the 119 policies reviewed, 37 (31%) were identified as having potential to impact systemically underserved groups and create or sustain inequities. Using our screening tool (Table 1), 26 (22%) screened in for question #1, 27 (23%) for question #2, and 31 (26%) for question #3. Most (n = 36; 97%) of these 37 policies with potential equity impact lacked specific existing equity considerations.

**Figure 1.**
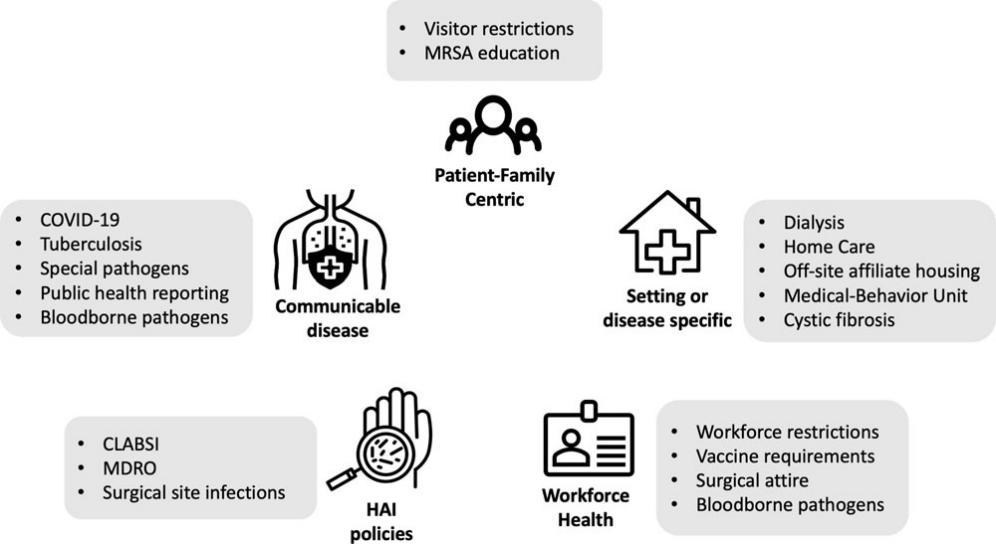
Examples of policy topic areas with potential to create or sustain inequities. Policies identified as having the potential to create or sustain inequities included five overarching categories with examples as shown.

The policies with potential equity implications covered the following five categories (Figure 1): (1) communicable diseases, (2) hospital-acquired infections (HAIs), (3) patient or family-centric, (4) disease or setting specific, and 5) occupational health. The policies included in the communicable diseases encompassed COVID-19 (including masking, workforce restriction, testing), tuberculosis, public health reporting guidance, bloodborne pathogen exposure management, and special pathogens program (including Ebola, Middle East Respiratory Syndrome, and Severe Acute Respiratory Syndrome). Within the hospital-acquired infection category, policies related to central-line associated bloodstream infections, multi-drug resistant organisms, and surgical site infections had potential equity implications. Policies in the patient or family-centric category included visitor restrictions and caregiver education policies relating to MRSA. Disease or setting-specific policies included those related to the medical behavioral unit, off-site affiliate housing, dialysis, home care, and cystic fibrosis care. Finally, occupational health related policies encompassed workforce member restrictions, bloodborne pathogen exposures, vaccine requirements, and surgical attire. Examples of policies that did not highlight inequities included those around facilities, construction, water intrusion, and transmission-based precautions. Policies that did not screen in for equity concerns were more likely to detail the use of equipment or procedures, and less likely to involve human factors.

The IP Equity Screen and related equity work have resulted in changes and improvements. For example, our team identified that multiple potential inequities existed within the care pathway for TB isolation and diagnosis, including potential bias in which patients may be considered at risk for TB infection or disease, potential inequities in the amount and level of detail of information families receive about the process, and how language-concordant care is delivered during this process, especially in the setting of airborne isolation. Furthermore, potential inequities can exist when considering caregiver, visitor, and sibling policies.^4^ These policies address some equity considerations by having a pathway for visitor exception requests and strategies for identifying social and cultural needs of families. We are working on ensuring these exception requests and needs are identified in a more standard manner. Additionally, we have expanded the translation of patient and family education material related to IP topics for patients and families who use a language other than English.

**Figure 2.**
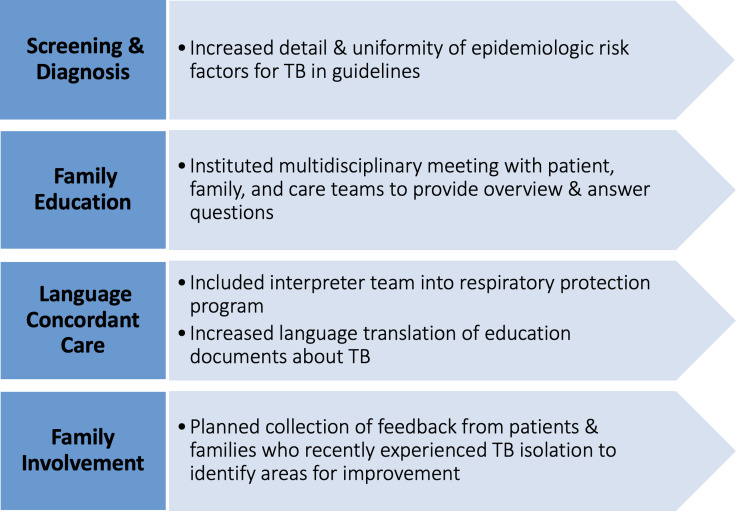
Tuberculosis policy changes after equity evaluation. Specific changes made to our organization’s approach to tuberculosis screening and diagnosis occurred at several process steps.

Most of our initial changes focused on TB-related guidelines, including standardizing how risk factors for TB are described in guidelines, creating a standard process with multidisciplinary huddle to better communicate TB isolation processes (including testing required for patients and families, visitor restrictions, and anticipated duration of isolation) to families in their language of care, and enhancing family involvement in process improvement (Figure 2).

## Discussion

Our single-center review of over 100 Infection Prevention policies for equity impacts demonstrates that such a review is feasible, and importantly that approximately one-third of our local IP policies possess the potential to impact those from historically underserved groups and create or sustain inequities. To our knowledge, our review is the first to describe the use of an EIA framework within the field of infection prevention.

Support from our organization’s content experts in diversity and health equity was crucial, given their guidance on key questions and considerations as well as next steps to take in response to potential inequities. Our local work has resulted in several changes to current policies and procedures in response to identified potential inequities, and additional work is ongoing.

Limitations to our work include that it took place at a single center, and thus some other organizations may need to adapt processes for generalizability to other settings. The identification of equity concerns can be context dependent and can depend on an individual’s positionality. Furthermore, though we created and used a standardized tool, policy review and the use of the tool may still be influenced by subjectivity and thus we could have failed to include all equity considerations or overestimated the equity impacts of some policies.

In summary, healthcare policies like those used in IP programs have the potential to create or sustain existing inequities. Systematic consideration of equity implications using an EIA framework and related tools could be the first step in mitigating these effects. Providing EIA tools applicable to infection prevention work could allow for dissemination of this practice more broadly within healthcare infection prevention programs. Concrete policy changes can be made in response to equity assessments to reduce potential inequities.

## References

[1] Bailey ZD , Krieger N , Agénor M , Graves J , Linos N , Bassett MT . Structural racism and health inequities in the USA: evidence and interventions. Lancet 2017;389:1453–1463. doi: 10.1016/S0140-6736(17)30569-X. 28402827

[2] Chen J , Khazanchi R , Bearman G , Marcelin JR . Racial/ethnic inequities in healthcare-associated infections under the shadow of structural racism: narrative review and call to action. Curr Infect Dis Rep 2021;23:17. doi: 10.1007/s11908-021-00758-x. 34466126 PMC8390539

[3] McGrath CL , Bettinger B , Stimpson M , et al. Identifying and mitigating disparities in central line-associated bloodstream infections in minoritized racial, ethnic, and language groups. JAMA Pediatr 2023;177:700–709. doi: 10.1001/jamapediatrics.2023.1379. 37252746 PMC10230370

[4] Olszewski AE , Adiele A , Patneaude A , Zerr DM , Kett JC . The health equity impact assessment: a case study in COVID-19 visitor policy. Hosp Pediatr. 2021;11(12):hpeds.2021-006128. doi: 10.1542/hpeds.2021-006128. 34737217

[5] Seattle Children’s Hospital Center for Diversity and Health Equity. Equity Impact Assessment. https://child.seattlechildrens.org/people_and_places/departments/center_for_diversity_and_health_equity/equity-impact-assessment/.

[6] New York State Department of Health. Health Equity Impact Assessment. https://www.health.ny.gov/community/health_equity/impact_assessment.htm. Accessed August 15, 2023.

[7] Povall SL , Haigh FA , Abrahams D , Scott-Samuel A . Health equity impact assessment. Health Promot Int 2014;29:621–633. doi: 10.1093/heapro/dat012. 23449601

[8] Michigan Department of Health and Human Services. Equity Impact Assessment (EIA). https://www.michigan.gov/mdhhs/-/media/Project/Websites/mdhhs/Doing-Business-with-MDHHS/Boards-and-Commissions/Coronavirus-Task-Force-on-Racial-Disparities/EIA-one-pager_MDHHS-Draft-2022.pdf?rev=2066310069394e0a8a00c46688f804a1&hash=E4BAA930C8F523A01C8E9D87D63CFCFE. Accessed August 15, 2023.

